# Current randomized control trials, observational studies and meta analysis in off-pump coronary surgery

**DOI:** 10.1186/s13019-015-0391-x

**Published:** 2015-12-17

**Authors:** Haralabos Parissis, Man Chi Lau, Mondrian Parissis, Savvas Lampridis, Victoria Graham, Reza Al-Saudi, Peter Mhandu

**Affiliations:** Cardiothoracic Department, Royal Victoria Hospital, Grosvenor Road, Belfast, BT12 6BA UK Ireland

**Keywords:** Off-pump coronary surgery, Coronary artery bypass surgery, beating heart surgery, RCTs in off-pump surgery, Off-pump versus on-pump

## Abstract

The off-pump literature is divided into three eras: the “early phase” with results favouring off-pump surgery supported with randomized control trials (RCTs) mainly from Bristol, UK; an “intermediate phase” dominated by the results of the ROOBY trial and finally a more “contemporary phase” whereby the off/on-pump argument is unsettled. Although the literature has failed to project an overall superiority of off-pump versus on-pump surgery, nevertheless, small randomized control trials and large meta-analysis studies are concluding that the incidence of a stroke is less than 1 % when an aortic off-pump techniques (especially the non-touch technique) are advocated in patients with diseased ascending aorta. Furthermore, off-pump combined with hybrid procedures may lead to a reduction of adverse outcome in the aged high-risk population with concomitant poor left ventricular function and co-morbidities.

The current review attempts to bring an insight onto the last ten years knowledge on the on/off-pump debate, with an aim to draw some clear conclusions in order to allow practitioners to reflect on the subject.

## Background

Most surgeons perform coronary bypass surgery with the aid of cardiopulmonary bypass, which (at least in theory) inflicts a massive systemic inflammatory response to the body, leading to adverse clinical outcome.

In an attempt to make coronary bypass less invasive, interest have been diverted to the off pump technique.

In the initial phase of off pump surgery, the procedure was performed without mechanical stabilizers and was borne out of cost containment. However, the off pump technique was technically challenging and limited to few surgeons only. With the introduction of mechanical stabilizers, off pump techniques are now technically less challenging and allow access to all sides of the heart for meticulous coronary anastomosis. Despite this, controversy still exists among the cardiac surgery community regarding the outcome, benefits and safety of this technique;

In this review, we invite the reader to take a stroll through the publishing knowledge on “off pump” coronary surgery; in this respect, we take the opportunity to examine and reflect on to what we have learnt from the current literature on the subject.

## Important Randomized Control Trials (RCTs)

There are only few RCTs through out the literature that compare on pump versus off pump coronary surgery. Angelini et al. [1] randomized 401 patients over a 2 year period (1997-1999); randomly allocated 200 patients to off-pump and 201 to on-pump coronary surgery. They excluded patients who had had myocardial infarction in the past month or who required grafting of the circumflex artery distal to the first obtuse marginal branch. In a subsequent study (BHACAS 2) [2] the previously excluded patients (from BHACAS 1) were also studied; the authors showed an improved short-term outcome with off-pump (reduced intubation time, ITU stay, inotropes requirements, blood loss/ transfusion requirements, AF, pneumonia, hospital stay) and similarly a favourable medium term outcome 1-3 years postoperatively. Overall, 10.8 % of grafts were occluded across both groups. Further, logistic regression analysis showed no evidence that grafts were more likely to be occluded in Off-pump than in On-pump patients.

The differences in MACE-free survival and quality of Life indicators did not approach statistical significance. Although these findings greatly bolstered the case of the proponents of Off-pump surgery, some shortcomings pointed out were that the modality of assessment of graft patency was non-invasive (Coronary multi-sliced CT) as against the gold standard of coronary angiography; also, the study was a single centre study by a single surgical team, and hence could not necessarily be extrapolated to other surgeons and centres. Another concern with those studies was the fact that only a small number of patients were enrolled; To tighten confidence limits and prove that off-pump is safer would require a significant number of patients to improve power. However, BHACAS remains one of the early important randomized series with the longest-follow up data available.

The long-term patency was studied with Coronary multi-sliced CT [3]. The likelihood of graft occlusion was no different between off-pump coronary artery bypass (10.6 %) and coronary artery bypass grafting with cardiopulmonary bypass (11.0 %) groups (odds ratio, 1.00; 95 % confidence interval, 0.55–1.81; P >0.99).

Nathoe et al. [4] randomized 281 low risk patients (only 1 or 2 vessel disease; excluded recent MI, poor LV) and found less transfusion requirements and less CKMB release. Clinical outcome was similar at 1 year. In a subgroup of patients where angiography was performed, patency at 1 year was similar, however off-pump was cost-effective: 14 % cheaper at 1 year.

Puskas et al. [5] randomized (SMART study) 200 unselected patients excluding reoperations, patients in cardiogenic shock or patients who had preoperative IABP insertion. Complete revascularization was achieved in both groups. There was shorter hospital stay, reduced blood loss and transfusion, reduced levels of cardiac enzymes (CKMB and TnI) in the off-pump group. Early hospital outcome was similar. This article highlights that completeness of revascularization was achievable in off-pump. Coronary angiography prior to hospital discharge on 93.4 % of enrolled patients was carried out and showed similar patency (99.0 % off-pump v. 97.7 % on-pump). Angiography at 1-year [6] in153 patients showed similar patency (93.6 % Off-pump v. 95.8 % On-pump). There was no difference in clinical outcome: Rates of death, stroke, myocardial infarction, angina, and re-intervention were similar at 30 days and 1 year. This paper serves as a useful reference for angiographic patency at 1 year. Furthermore in a late follow up by the same authors [7] 190 grafts assessed by computed tomographic angiography with comparable patency. At late follow-up, recurrent angina had occurred in 25.6 % of off pump patients and 11.4 % of the on pump patients (p = 0.09).

Legare et al. [8] randomized 300 patients with EF > 30 % and found no differences in mortality, MI, stroke, transfusion, Atrial Fibrillation, time to extubation, ITU stay and hospital stay. This paper serves as a useful reference for the possible “lack of beneficial outcome” of off-pump coronary surgery, especially in low-risk patients.

Angiographic outcomes of Off pump surgery were reported by Al-Ruzzeh et al. [9] on a randomized population of 168 patients; Graft patency was evaluated by angiography at 3 months and was similar between the on-pump and off-pump groups. Patients in the off-pump group required fewer blood transfusions (1.7 units v 1.0 unit, P = 0.02), shorter duration of mechanical ventilation (7.7 hours v 3.9 hours, P = 0.03), and shorter hospital stay (10.8 days v 8.9 days). Scores for neurocognitive function showed a significant difference in three memory subtests at six weeks and two memory subtests at six months in favour of the off-pump group.

The ROOBY trial [10] recruited 2203 patients randomized to on versus off–pump (on-pump (n = 1,099) or off-pump (n = 1,104) procedures). It is noteworthy that this trial is by far the largest RCT comparing the effects of on and off pump surgery.

The primary short-term end point was a composite of death or complications (reoperation, new mechanical support, cardiac arrest, coma, stroke, or renal failure) before discharge or within 30 days after surgery. The primary long-term end point was a composite of death from any cause, a repeat revascularization procedure, or a nonfatal MI within 1 year after surgery. Secondary end points included the completeness of revascularization, graft patency at 1 year, neuropsychological outcomes, and the use of major resources.

The authors reported no significant difference in 30-day mortality between the off-pump group and the on-pump group (2 % [18/1104] and 1 % [13/1099] respectively, p = 0.47). The proportion of patients with fewer grafts than originally planned, was higher with off-pump CABG than with on-pump CABG (17.8 % vs. 11.1 %, P < 0.001). Follow-up angiograms in 1371 patients who underwent 4093 grafts revealed that the overall rate of graft patency was lower in the off-pump group than in the on-pump group (82.6 % vs. 87.8 %, P < 0.01). There were no differences in cognitive function, as well as no neurological differences were noted between the two groups at one year. Survival post surgery up to 2000-days was slightly lower in the off pump treatment group.

The ROOBY trial received criticisms: firstly, the patients enrolled were almost exclusively males; secondly there was a trend toward enrolling lower-risk patients and excluding higher-risk.

During trial recruitment period, 9663 patients who were scheduled for urgent or elective CABG were screened for enrolment, 7460 patients (77.2 %) were excluded; 2716 patients because of diffuse disease or small coronary arteries, 2461 because of unavailability of a participating surgeon or study coordinator, or patient refusal or inability to participate in 1467 patients.

Lastly, the ROOBY trial was criticized for the fact that the conversion rate to Cardio pulmonary bypass was unacceptably high at 12 % and this brought some scepticism on the level of “off pump” experience of the surgeons involved in the study.

An RCT on high risk patients, defined as EuroSCORE ≥5 undergoing on versus off-pump surgery was reported by Moller et al. ([11]. 341 patients with 3-vessel coronary disease were recruited. The primary outcome was a composite of adverse cardiac and cerebrovascular events. Exclusion criteria were previous heart surgery, left ventricular ejection fraction <30 %, lack of informed consent, and unstable preoperative condition. Fewer grafts were performed to the lateral part of the left ventricle territory during off-pump surgery (0.97 versus 1.14 after on-pump surgery; *P* = 0.01). Eight patients (4.5 %) allocated to off-pump CABG crossed over to on-pump CABG. There were no significant differences in the composite primary outcome (15 % versus 18 %; *P* = 0.48). The 3-year follow up [12] of this trial showed that MACCE occurred in 40 % patients allocated to off-pump versus 33 % of the patients allocated to on-pump CABG. All-cause mortality was significantly increased in the off-pump group (24 % versus 15 %; HR 1.66, 95 % CI 1.02 to 2.73; p = 0.04), but cardiac-related death was not significantly different (10 % versus 7 %; HR 1.30, 95 % CI 0.64 to 2.66; p = 0.47). The authors concluded that the mortality was higher following off-pump surgery. However, this trial cannot provide any answers on the potential benefit or harm that off-pump coronary artery bypass graft surgery may have on patients with ejection fraction <30 %, those who are undergoing redo coronary artery bypass graft surgery, or those patients in an unstable preoperative condition/emergency operation. With other words, the patients recruited in this trial were marginally high-risk population and therefore the lack of significant difference in the outcome was predictable; furthermore an-aortic technique was not employed in this group of patients.

Houlind et al. [13] (The DOORS trial) set off to determine graft patency between on and off pump surgery, with post operative angiography: In a randomized, controlled, multicenter trial, 900 patients more than 70 years of age received either on-pump or off-pump coronary artery bypass surgery. A total of 481 patients underwent angiography. In the off-pump group, 561 (79 %) of 710 grafts were open, 65 (9 %) were stenotic, and 84 (12 %) were occluded. In the on-pump group, 549 (86 %) of 650 grafts were open, 38 (5 %) were stenotic, and 63 (9 %) were occluded. The difference between the proportions of open grafts was statistically significant in favour of on-pump surgery (P = .01).

The future is exciting with a number of RCTs on the way:

CORONARY [14] is the largest RCT yet conducted comparing off-pump CABG to on-pump CABG. The plan was to randomize 4,700 patients to on or off pump. The co-primary outcomes are a composite of total mortality, myocardial infarction (MI), stroke, and renal failure at 30 days and a composite of total mortality, MI, stroke, renal failure, and repeat revascularization at 5 years. As of May 3, 2011, CORONARY has recruited 3,884 patients from 79 centres in 19 countries. Currently, patient’s mean age is 67.6 years, 80.7 % are men, 47.0 % have a history of diabetes, 51.4 % have a history of smoking, and 34.4 % had a previous MI. In addition, 20.9 % of patients have a left main disease, and 96.6 % have double or triple vessel disease. Finally 4752 patients were recruited [15], and early results published in 2012. There was no significant difference between off-pump and on-pump CABG with respect to the rate of death, nonfatal myocardial infarction, nonfatal stroke or renal failure requiring hemodialysis at 30 days after randomization. Subgroups analysis of 244 patients with ejection fraction between 20-34 % showed that there was no significant difference concerning the co-primary outcome (Hazard Ratio: 0.91(0.47-1.74), p = 0.77).

However, the off-pump techniques significantly reduced the rate of blood-product transfusion (50.7 % vs 63.3 %; P < 0.001), reoperation for perioperative bleeding (1.4 % vs 2.4 %; P = 0.02), acute kidney injury (28.0 % vs 32.1 %; P = 0.01), and respiratory complications (5.9 % vs 7.5 %; P = 0.03) but increased the rate of early repeat revascularizations (0.7 % vs 0.2 %; P = 0.01).

The authors attributed the overall encouraging short-term benefits to the high level of expertise and technical skills for participating surgeons and their abilities to satisfactorily manage the inherent difficulties in performing delicate anastomoses on a beating heart and yet attain a near-equal level of complete revascularization in the off-pump surgery group as compared with the on-pump surgery group (88.2 % vs 90 %, respectively; P = 0.05).

At 1 year [16], there was no significant difference in the rate of the primary composite outcome between off-pump and on-pump CABG (12.1 % and 13.3 %, respectively; hazard ratio with off-pump CABG, 0.91; 95 % confidence interval [CI], 0.77 to 1.07; P = 0.24). The rate of the primary outcome was also similar in the two groups in the period between 31 days and 1 year (hazard ratio, 0.79; 95 % CI, 0.55 to 1.13; P = 0.19). The rate of repeat coronary revascularization at 1 year was 1.4 % in the off-pump group and 0.8 % in the on-pump group (hazard ratio, 1.66; 95 % CI, 0.95 to 2.89; P = 0.07). There were no significant differences between the two groups at 1 year in measures of quality of life or neurocognitive function.

The GOPCABE trial [17] had randomly assigned patients 75 years of age or older who were scheduled for elective first-time CABG on or off pump. The primary end point was a composite of death, stroke, myocardial infarction, repeat revascularization, or new renal-replacement therapy at 30 days and at 12 months after surgery. A total of 2539 patients underwent randomization. At 30 days after surgery, there was no significant difference between patients who underwent off-pump surgery and those who underwent on-pump surgery in terms of the composite outcome (7.8 % vs. 8.2 %; odds ratio, 0.95; 95 % confidence interval [CI], 0.71 to 1.28; P = 0.74) or four of the components (death, stroke, myocardial infarction, or new renal-replacement therapy). Repeat revascularization occurred more frequently after off-pump CABG than after on-pump CABG.

The criticism to this trial includes several points:

1. The need for intra operative graft assessment, since it is known that surgical revision may be necessary in certain cases. Such assessment is not mentioned in this report. In addition, antithrombotic management is not reported. This lack of standardization in the off-pump CABG group is a limitation and may explain why repeat revascularization was the only significant difference (1.3 % vs. 0.4 %; odds ratio, 2.42; 95 % CI, 1.03 to 5.72; P = 0.04).

The GOPCABE trial goes on to report that at 12 months, there was no significant between-group difference in the composite end point (13.1 % vs. 14.0 %; hazard ratio, 0.93; 95 % CI, 0.76 to 1.16; P = 0.48) or in any of the individual components. Similar results were obtained in a per-protocol analysis that excluded the 177 patients who crossed over from the assigned treatment to the other treatment.

2) The authors should have presented an “as-treated” analysis (not as intention to treat) based on the operation actually performed, and the patients who crossed over from the assigned treatment should not have been excluded.
3) 37 high-risk patients crossed over to off-pump CABG because of a calcified ascending aorta, the stroke rate was still high among patients who underwent off-pump CABG (2.2 %). It is established that systematic application of “anaortic” techniques (i.e., off-pump CABG without aortic manipulation) reduces the rate of stroke to less than 1 %, 2-4 but no information about aortic management was provided.

The MASS III trial [18] was a single-centre study that evaluated 308 patients with multivessel coronary artery disease randomized to on-pump (153) or off-pump (155) CABG. Of this total, 260 (84.4 %) patients had, on coronary angiography, at least one 70 % obstruction in the circumflex territory (141 on-pump and 119 off-pump). The combined outcome was death, myocardial infarction, target vessel revascularization (angioplasty or surgery) or hospitalization for cardiac causes. After 5 years of follow-up, off-pump CABG had higher combined events than on-pump had: 25 (21 %) versus 17 (12 %), hazard ratio 1.88, 95 % confidence interval 1.02-3.48, P = 0.041. In the multivariate model with the inclusion of the following variables: age (P = 0.09) and complete revascularization (P = 0.68), off-pump surgery remained as a predictor of combined events in 5 years, P = 0.03.

Treatment impact on diabetic patients (in 835 patients at the ROOBY trial) randomized to received off-pump CABG (n = 402) or on-pump CABG (n = 433) was reported by Shroyer et al. [19]. For diabetic patients, the primary short-term composite outcome rate showed a worse trend for off-pump (8.0 %) than on-pump (3.9 %, p = 0.013), with no difference in the 1-year primary composite outcome or 1-year death rate. One-year patency was 83.1 % off-pump versus 88.4 % on-pump (p = 0.004). No differences were found in neurocognitive, health-related quality of life, discharge cost, and 1-year cumulative cost.

Finally, only extended follow-up of existing randomized trials and new well-designed randomized trials together with cumulative meta-analysis of such trials will yield robust evidence on the off-on pump argument.

## “Important” Large Observational studies

Cleveland et al. [20] reported the early experience of the STS National Database from 1998 - 1999. In that analysis, 106,423 patients underwent on-pump surgery and 11,717 patients underwent off-pump. The latter group was more likely to be older, female, and have more chronic obstructive pulmonary disease and renal failure. On-pump patients were more likely to be diabetic, have 3-vessel or left main disease, or require emergency operation. Risk-adjusted mortality favoured off-pump (2.31 % versus 2.93 %, P = 0.0001).

Off-pump patients had fewer strokes (2.5 % versus 4.6 %, P =0.0001), less prolonged mechanical ventilation (8.9 % versus 11.3 %), less re-exploration for bleeding (2.1 % versus 2.8 %), and shorter postoperative length of stays (6.1 versus 7.0 days).

A report of the New York State Database [21] from 1997 to 2000 analyzed 59,044 on-pump and 9135 off-pump patients. There was significantly greater proportion of older patients, women patients with previous stroke as well as renal failure, in the off-pump group. Even though the risk-adjusted operative mortality rate for off-pump was lower (2.02 % versus 2.16 %) it did not reach statistical significance. Off-pump patients did have less stroke rate (1.6 % versus 2.0 %, P = 0.0003), and re-operation for bleeding (1.6 % versus 2.2 %, P = 0.0001), as well as shorter postoperative length of stay (5 versus 6 days, P = 0.0001). Improved survival at 3-year follow-up in the on-pump group (89.5 % versus 88.8 %, P = 0.022) may have been affected by the older, sicker off-pump patient’s profile.

Mack et al. [22] published a large retrospective series consisting of 17,548 patients with multi-vessel disease (from 4 centres over a 3-year period) matched by propensity scoring (7200 off pump Vs. 11000 on pump). In this study, the authors found that the mortality was significantly less in the off-pump coronary artery bypass-grafting group (2.8 % versus 3.7 %, P <0.001). They concluded that the use of CPB was a predictor of overall mortality (OR 2.08) and also of mortality in re-operations (OR 3.37), female patients (OR 1.7) and patients over 75 years (OR 2.13). Furthermore, they also reported that off-pump is associated with reduced short-term morbidity reductions in blood transfusion (32.6 % versus 40.6 %, P <0.001), stroke (1.4 % versus 2.1 %, P =0.002), renal failure (2.6 % versus 5.2 %, P < .001), pulmonary complications (4.1 % versus 9.5 %, P < .001), re-operation (1.7 % versus 3.2 %, P < .001), atrial fibrillation (21.1 % versus 24.99 %, P < .001), and gastrointestinal complications (3.6 % versus 4.8 %, P = .02). ). This was a short-term observational study with no mid/long term follow up. However, this serves as a good reference for potential usefulness of off-pump in re-operations and elderly patients.

Hannan et al. [23] reported on New York State patients who underwent either off-pump (13 889 patients) or on-pump CABG surgery (35 941 patients) between 2001 and 2004. An experience off-pump surgeon performed two thirds of the off-pump cases. A total of 226 patients (1.63 %) were converted from off pump to on-pump CABG, and their in-hospital mortality rate was 9.73 %. Off pump had a significantly lower inpatient mortality rate (adjusted OR 0.81) and lower rates for two peri-operative complications (stroke: adjusted OR 0.70, 95 % CI 0.57 to 0.86; and respiratory failure: adjusted OR 0.80, 95 % CI 0.68 to 0.93). However off pump was associated with lower freedom from subsequent revascularization, 89.9 % Vs. 93.6 % (*P* < 0.0001).

A large non-randomized trial was reported at the STS, 2009 [24]. The authors used the power of the STS database to provide direct comparison between on-pump and off- pump surgery. To remove the bias of low-volume centre or surgeons and allow a critical look at the results of each technique in relatively experienced hands, they limited the analysis to centres that performed at least 150 off-pump and 150 on-pump cases over a 3-year period. This resulted in a study group of 186,458 patients. Of that cohort, 65,864 underwent off-pump, whereas 120,594 underwent on-pump surgery. They further analyzed patients by coronary anatomy, grouping patients with 1 or 2 diseased vessels (45,969 patients), 3 diseased vessels without left main stenosis (97,997 patients), and 3 diseased vessels with left main stenosis (42, 492 patients). A multivariate logistic analysis of 32 risk factors was used to risk-adjust outcomes. Importantly, crossovers from off-pump to on-pump were analyzed on an intention-to-treat basis. The off-pump group was more likely to be elderly people, women, and have suffered a preoperative stroke or renal failure, whereas the on-pump group were more likely to be diabetic, have a low ejection fraction congestive heart failure, more single and 2 vessel disease (34 % versus 19.6 %, P = <0.001), therefore requiring fewer grafts (3.04 versus 3.58, P = 0.001). The authors show a significant reduction in operative mortality in the off-pump group, as well as a highly significant reduction in overall adverse cardiac events, permanent stroke, dialysis, re-operation, prolonged ventilation, sternal wound infection, renal failure, and prolonged length of stay. These results were consistent across anatomic groups, whether patients had 1-2 vessels diseased, or 3 diseased vessels with or without left main stenosis.

## “Important” Meta-analysis

Wijeysundera et al. [25] reported on 37 randomized controlled trials (n = 3,449 patients) and 22 risk-adjusted (logistic regression or propensity-score) observational studies (n = 293,617). The overall conversion rate was high at 13 %.

When the authors studied closely the 37 RCTs, they found that off-pump was associated with reduced atrial fibrillation (OR 0.59) and trends toward reduced 30-day mortality (OR 0.91), stroke (OR 0.52), and myocardial infarction (OR 0.79).

The analysis of the Observational studies showed, off-pump to be associated with reduced 30-day mortality (OR 0.72), stroke (OR 0.62), infarction (OR 0.66), and atrial fibrillation (OR 0.78). However, only atrial fibrillation reached statistically significance reduction in off pump group in both RCTs and observational studies. One can be critical when observing that the difference in the incidence of stroke changed from statistically significant to non-significant, when compare results from observational studies to RCTs. This difference in findings points to selection bias as a source of systematic error in the meta-analyses that included observational studies.

Finally at one to two years, off-pump was associated with increased incidence of repeat revascularization.

Moller et al. [26] reported on a meta-analysis of 66 randomized trials of off versus on pump, including 5202 patients. There were no statistically significant differences between the two groups, regarding mortality [relative risk (RR) 0.98; 95 % confidence interval (CI) 0.66–1.44], myocardial infarction (RR 0.95; 95 % CI 0.65–1.37), or repeat coronary revascularization (RR 1.34; 95 % CI 0.83–2.18). There was a significant reduced risk of atrial fibrillation (RR 0.69; 95 % CI 0.57–0.83) in the off-pump group.

Subsequently Moller [27] looked at 10 “low-bias” trials (n = 4950) of a total of 86 RCTs comparing Off-pump with On-pump surgery. The authors showed that off-pump CABG increased all-cause mortality compared with on-pump CABG; the effect was more pronounced in the trials at low risk of bias: (6.2 %) off-pump versus (4.6 %) on-pump, RR 1.35,95 % CI 1.07 to 1.70; P = .01). This translates to a 30 % higher risk of all-cause mortality after off-pump CABG compared with on-pump CABG.

Other adverse events like MI, stroke, renal insufficiency or repeat revascularization were similar despite a slightly lower number of distal anastomoses Off-pump versus On-pump. The report criticised, by the fact that studies with high mortality following off pump surgery, were included in this meta-analysis.

Feng et al. [28] reported on a meta-analysis of ten randomized trials (2,018 patients) of off-pump versus on-pump surgery. The primary outcome was defined as all-cause mortality, stroke, myocardial infarction, and revascularization at 1 year. Off-pump coronary artery bypass surgery in this study did not differ from on-pump surgery with regard to mortality, rates of stroke, myocardial infarction, or revascularization.

Tagaki et al. [29] performed an updated meta-analysis of graft patency after off-pump versus on-pump CABG from randomized trials. This meta-analysis included data on 6898 grafts and suggests that off- pump CABG may increase overall graft occlusion by 32 %, over on-pump CABG.

The same group [30] went on to look as to whether off versus on pump techniques have any influence on mid term MACCE. Eight RCTs enrolling 10 954 patients were identified and included. A pooled analysis demonstrated no statistically significant difference in off-pump and on-pump CABG (hazard ratio, 1.10; 95 % confidence interval, 0.93-1.29; P = 0.27). The authors concluded that off-pump CABG appears not to increase mid-term MACCE over on-pump CABG.

Attaran et al. [31] reported in a meta analysis looking into whether off-pump coronary artery bypass improves outcomes when compared to on-pump coronary artery bypass in the female population. A systematic literature review identified six observational studies, incorporating 23313 patients (n = 9596 OPCAB, 13717 ONCAB). 30-day mortality was similar between the two techniques therefore the authors concluded that off pump is a safe alternative to on pump in the surgical revascularisation of female patients.

Chen et al. [32] did a meta-analysis of 43 randomized clinical trials with 8104 patients in the off-pump group and 8724 cases in the on-pump group. The meta-analyses of these trials showed no significant difference between off-pump and on-pump in the incidences of stroke (odds ratio (OR) = 0.80, 95 % confidence interval (CI) = 0.52 - 1.22, P = 0.30) and MI (OR = 0.73, 95%CI = 0.52 - 1.02, P = 0.06). However, there was a significantly reduced risk of AF (OR = 0.65, 95%CI = 0.52 - 0.82, P = 0.0002) in off-pump patients.

Godinho et al. [33] reported on nine randomized clinical trials, corresponding to a total of 75,086 patients, and compared off-pump to on-pump. A reduction of 18 % in the risk of cardiovascular mortality (OR: 0.82, 95%CI: 0.70 to 0.98, p = 0.03) and 27 % in the risk of stroke postoperatively (OR: 0.73, 95%CI: 0.63 to 0.85, p = 0.0001) were observed, both in favor of off-pump. Concerning the occurrence of complications associated with the procedure, no significant differences were found between the two surgical techniques, particularly with regard to the occurrence of renal complications (OR: 0.97, 95%CI: 0.84-1.14, p = 0, 74) and sepsis (OR 0.98, 95%CI: 0.64-1.51, p = 0.93, respectively).

An important meta-analysis, using propensity score analyses, reported by Kuss et al. [34]; 123,137 patients were included in this report. The estimated overall odds ratio was less than 1 for all outcomes, favouring off-pump surgery. This benefit was statistically significant for mortality (odds ratio 0.69; 95 % confidence interval, 0.60–0.75), stroke, renal failure, red blood cell transfusion (P < .0001), wound infection (P < .001), prolonged ventilation (P <0.01), inotropic support (P < .02), and intra-aortic balloon pump support (P < .05). The odds ratios for myocardial infarction, atrial fibrillation, and re-operation for bleeding were not significant.

A much smaller meta-analysis of randomized trials by Takagi and colleagues [35] focused on a late (>1 year) all caused mortality of off-pump versus on pump; the authors concluded that off-pump may increase late all-cause mortality by a factor of 1.37 over on-pump CABG.

A meta-analysis published by Afilalo et al. [36] included all published and unpublished RCTs of Off-pump versus On-pump CABG from the MEDLINE, EMBASE and Cochrane databases. This report, took into consideration a total of 59 trials, encompassing 8961 patients. There was a significant 30 % reduction in post-operative strokes with Off-pump surgery [risk ratio (RR) 0.70, 95 % CI: 0.49–0.99]. There was no significant difference in mortality (RR: 0.90, 95 % CI: 0.63–1.30) or MI (pooled RR: 0.89, 95 % CI: 0.69–1.13). In the meta-regression analysis, the effect of Off-pump on all of the clinical outcomes was similar regardless of mean age, proportion of females in the trial, number of grafts per patient, and trial publication date.

Sa et al. [37] looked and reported at Forty-seven RCTs included 13,524 patients (6,758 for off-pump and 6,766 for on-pump CABG). There was no significant difference between off-pump and on-pump CABG groups in RR for 30-day mortality or myocardial infarction. However Off-pump CABG found to reduce the incidence of post-operative stroke by 20.7 %.

Table 1, is depicting the most important publications on Off-Pump vs. On-pump argument; the reported studies are matched with the Level of evidence and key results of each individual study is given.

**Table 1:** The most important publications on Off-Pump vs. On-pump, matched with the appropriate “Level of evidence” and “key results” of each individual study

Author Patient Group, study type/Level of Evidence Outcomes & Key Results		
Important Randomized Control Trials (RCTs)		
[1] Angelini G et al, Lancet (2002)	RCT, (BHACAS 1)	Less AFib, Inotrop use, Transfusions and hospital stay in Off-pump group. The authors concluded that Off-pump coronary surgery significantly lowers in-hospital morbidity without compromising outcome in the first 1-3 years after surgery compared with on-pump surgery.
	200 pts Off pump vs. 201 pts on pump	
	From 1997 to 1999. Operations performed by experience surgeons.	
	Aim to assess mortality and cardiac related events at mid-term follow-up (25 months).	
[2] Ascione R et al, European heart journal (2004)	RCT, (BHACAS 2)	Both groups showed a similar deterioration of the “quality of life scoring systems” with time.
	200 pts Off pump vs. 201 pts on pump	
	Aim to assess disease specific quality of life at mid-term follow up.	
[3] Angelini G et al, J Thorac Cardiovasc Surg (2009)	6 to 8 years follow up of the BHACAS pts.	The likelihood of graft occlusion was no different between off-pump coronary artery bypass (10.6 %) and coronary artery bypass grafting with cardiopulmonary bypass (11.0 %) groups (odds ratio, 1.00; 95 % confidence interval, 0.55-1.81; P > .99).
	Aim to assess graft patency (multisliced CT) and MACCE.	
		There were no differences between off-pump and on pump groups in the hazard of death (hazard ratio, 1.24; 95 % confidence interval, 0.72-2.15) or MACCE (hazard ratio, 0.84; 95 % confidence interval, 0.58-1.24)
[4] Nathoe HM et al, N Engl J Med (2003)	Multicenter RCT.	Graft patency was similar and above 90 % in both groups. Freedom from MACCE was 90 %, somehow similar between the two groups. At 1 year off pump was $1,839 cheaper per pt studied.
	Low risk patients predominantly single or double vessel disease. 140 pts either arm of the study.	
	Graft patency, MACCE and cost-effectiveness in 1 year following surgery, was reported.	
[5] Puskas J et al, J Thorac Cardiovasc Surg (2003)	Single-centre RCT.	Number of grafts performed and index of completeness of revascularization were similar.
	200 unselected, elective CABGs were randomly assign to Off vs. On pump.	Off pump group has less myocardial enzyme rise, less coagulopathy, less transfusion requirements and shorter intubation time.
	From 2000 to 2001.	
	Operations performed by experience surgeons.	
[6] Puskas J et al, JAMA (2004)	1 year Follow-up of the above RCT.	Similar graft patency and MACCE between the 2 groups at 30 days and 1 year. Off pump appeared to be cost-effective.
	Graft patency, MACCE and cost-effectiveness, in 30 days and 1 year following surgery was reported.	
[7] Puskas J et al, Ann Thorac Surg (2011)	7.5 year Follow-up of the above RCT.	Mortality, around 30 % at 7.5 years in both groups.
	Early graft patency was assessed with angiography and late with Multisliced CT.	Graft patency, around 80 % at 7.5 years in both groups.
	Late graft patency, recurrence of ischemia and need for re intervention was reported.	Re intervention rate 2.3 % at 7.5 years in both groups.
[8] Legare JF et al, Circulation (2004)	Single-centre RCT.	Excellent postoperative results without significant differences were demonstrated with either procedure.
	300 pts divided into two equal arms. Pts with EF < 30 % were excluded.	
	Postoperative morbidity and mortality was compared between the two groups.	
[9] Al-Ruzzeh et al, BMJ (2006)	Single-centre RCT.	Similar graft patency between the two groups at 3 months.
	168 pts divided into two equal arms.	
	Angiographic examination was carried out at three months postoperatively. Neurocognitive tests were carried out at baseline and at six weeks and six months postoperatively.	Interestingly, Scores for neurocognitive function showed a significant difference in three memory subtests at six weeks and two memory subtests at six months in favor of the off-pump group.
[10] ROOBY Trial, N Engl J Med (2009)	Multi-centre RCT.	Similar 30-day composite outcome around 6-7 %. One year composite outcome 9.9 % off-pump vs. 7.4 % on-pump, P = 0.04.
	2203 pts randomly assign to either treatment.	
	Composite of death and complications within 30 days and in 1 year following surgery was investigated.	Off-pump pts had fewer grafts than originally planned.s
	The surgeons experience was questioned. The conversion to on pump rate was also questioned.	Early follow-up angiograms showed patency of 82.6 % off-pump vs. 87.8 % on-pump, P < 0.01.
[11] Moller CH et al, Circulation (2010)	Single-centre RCT.	Fewer grafts were performed to the lateral part of the LV wall during off-pump surgery (0.97 versus 1.14 after on-pump surgery; P = 0.01).
	30-day outcome in high risk, three-vessel disease, patients (EuroSCORE > or = 5).	
	Interestingly, pts with EF < 30 % were excluded!	No significant differences in the composite primary outcome (15 % vs. 17 %; P = 0.48) or the individual components were found at 30-day follow-up
	341 pts randomly assign to either treatment.	
	Primary outcome was 30-day mortality and MACCE.	
[12] Moller CH et al, Heart (2011)	3.5 years follow-up of the previous RCT.	All-cause mortality was significantly increased in the off-pump group (24 % vs. 15 %; HR 1.66, 95 % CI 1.02 to 2.73; p = 0.04), but cardiac-related death was not significantly different (10 % vs. 7 %; HR 1.30, 95 % CI 0.64 to 2.66; p = 0.47). I am wondering, if that reflects a sicker general population in the off-pump group!
	Primary comparative outcome was intermediate mortality and MACCE.	
[13] Houlind et al, J Thorac Cardiovasc Surg. (2014)	DOORS Trial.	The proportion of open left internal thoracic artery grafts was 95 % in both groups. However, vein graft patency after off-pump surgery was inferior to that after on-pump surgery.
	Multicenter RCT. 900 patients randomized to On versus Off pump. 481 patients underwent angiography post operatively.	
[14] Lamy A et al, American Heart Journal (2012)	CORONARY Trial	The primary short-term end point was a composite of death or complications (reoperation, new mechanical support, cardiac arrest, coma, stroke, or renal failure) before discharge or within 30 days after surgery. The primary long-term end point was a composite of death from any cause, a repeat revascularization procedure, or a nonfatal MI within 1 and 5 year after surgery. Secondary end points included the completeness of revascularization, graft patency at 1 year and neuropsychological outcomes.
	Multicenter RCT. 4752 patients randomized to On versus Off pump.	
[15] Lamy A et al, N Engl J Med (2012)	CORONARY Trial.	No significant difference in the rate of the primary composite outcome. The authors concluded that although there was no significant difference between Off pump and on-pump CABG with respect to the 30-day mortality, MI, stroke, or renal failure requiring dialysis, the use of OPCAB resulted in reduced rates of transfusion, reoperation for perioperative bleeding, respiratory complications, and acute kidney injury at the expense of an increased risk of early revascularization.
	Early report of 30 days primary outcomes	
[16] Lamy A et al, N Engl J Med (2013)	CORONARY Trial.	No difference in the rate of the primary composite outcome between off-pump and on-pump CABG
	1 year results	
[17] Diegeler A et al, N Engl J Med (2013)	GOPCABE Clinical Trial. 2539 patients above 75 years old, randomized to on versus off pump. The primary end point was a composite of death, stroke, myocardial infarction, repeat revascularization, or new renal-replacement therapy at 30 days and at 12 months after surgery.	There was no significant difference between the two groups, regarding the primary end points. Repeat revascularization occurred more frequently after off-pump CABG than after on-pump CABG (1.3 % vs. 0.4 %; odds ratio, 2.42; 95 % CI, 1.03 to 5.72; P = 0.04). points.
[18] Vieira de Melo RM et al, Eur J Cardiothorac Surg (2014)	5 year follow up of the MASS III TRIAL. Single Centre RCT that evaluates 308 patients: on-pump (153 pts) and off-pump (155pts).	The authors concluded that in patients with multivessel coronary artery disease, off-pump coronary artery bypass surgery resulted in a higher incidence of cardiac events at 5-year follow-up.
[19] Shroyer AL et al. Ann Thorac Surg (2014)	Diabetic subgroup of the ROOBY Trial. 835 patients: 402 pts received off-pump CABG and 433 pts received on-pump CABG.	The 1-year graft patency was lower and the short-term composite adverse outcome was higher on the off-pump CABG group.
Large Observational studies		
[20] Cleveland JC et al, Ann Thorac Surg (2001)	STS Database.	Operative mortality 2.3 % for off-pump vs. 2.9 % with on-pump, P < 0.001.
	From 1998 to 1999. 126 experience centres.	
	118140 CABGs with 11.717 Off-pump cases.	MACCE 10.62 % for off-pump vs. 14.15 % with on-pump, P < 0.001.
[21] Racz MJ et al, Journal Of American College Of Cardiology (2004)	CABG surgery from 1997 to 2000 in the state of New York.	Mortality was 2.02 % for off-pump vs. 2.16 % for on-pump (p = 0.390)
	59044 on-pump pts vs. 9135 off-pump pts.	Off-pump patients had lower rates of perioperative stroke (1.6 % vs. 2.0 %, p = 0.003)
	The study compare in-hospital mortality and complications and 3-year mortality.	On-pump patients experience better long-term survival and freedom from revascularization than off-pump patients. However, the survival benefit from on-pump procedures was no longer present in the last two years of the study.
[22] Mack M et al, J Thorac Cardiovasc Surg (2004)	4 centres over 3-year period: 7283 off-pump pts vs.10.118 on-pump.	Mortality was significantly less in the off-pump coronary artery bypass grafting group (2.8 % vs. 3.7 %, P < .001).
	The study compare in-hospital mortality and complications.	Off-pump was associated with reductions in blood transfusion (32.6 % vs. 40.6 %, P < .001), stroke (1.4 % vs. 2.1 %, P = .002), renal failure (2.6 % vs. 5.2 %, P < .001), pulmonary complications (4.1 % vs. 9.5 %, P < .001), reoperation (1.7 % vs. 3.2 %, P < .001), atrial fibrillation (21.1 % vs. 24.99 %, P < .001).
[23] Hannan EL et al, Circulation (2007)	New York Database over 4 years period. 13889 off-pump vs. 35941 on-pump pts.	Off-pump had a significantly lower 30-day mortality rate (adjusted OR 0.81, 95 % confidence interval [CI] 0.68 to 0.97) and lower rates for 2 complications (stroke: adjusted OR 0.70, 95 % CI 0.57 to 0.86; respiratory failure: adjusted OR 0.80, 95 % CI 0.68 to 0.93).
		What became important in this study was that
		Off-pump patients had higher rates of subsequent revascularization (hazard ratio 1.55, 95 % CI 1.33 to 1.80). This could be potentially explained by the fact that Off-pump pts may have fewer grafts than originally planned.
	Postoperative and 3 years outcomes compared between the two groups.	
[24] Puskas J et al, 45^th^ STS Annual Meeting (2009)	STS database.	There was a significant reduction in operative mortality in the off-pump group, as well as a highly significant reduction in overall adverse cardiac events in this group.
	65,864 underwent off-pump, whereas 120,594 underwent on-pump surgery.	
Important Meta-analysis		
[25] Wijeysundera DN et al, Journal Of American College Of Cardiology (2005)	A meta-analysis of 37 randomized controlled trials (RCTs) (n = 3,449) and 22 risk-adjusted (logistic regression or propensity-score) observational studies (n = 293,617).	In RCTs, Off-pump was associated with reduced atrial fibrillation (OR 0.59; 95 % CI 0.46 to 0.77) and trends toward reduced 30-day mortality (OR 0.91 95 % CI 0.45 to 1.83), stroke (OR 0.52; 95 % CI 0.25 to 1.05), and myocardial infarction (OR 0.79; 95 % CI 0.50 to 1.25).
		Observational studies showed off-pump to be associated with reduced 30-day mortality (OR 0.72; 95 % CI 0.66 to 0.78), stroke (OR 0.62; 95 % CI 0.55 to 0.69), infarction (OR 0.66; 95 % CI 0.50 to 0.88), and atrial fibrillation (OR 0.78; 95 % CI 0.74 to 0.82).
		At 2 years off-pump was associated with increase repeat revascularization procedures.
[26] Moller CH et al, Eur Heart Journal (2008)	Meta-analysis of 66 randomized trials published up till 2007.	Off-pump was associated with a significant reduced risk of atrial fibrillation (RR 0.69; 95 % CI 0.57-0.83).
[27] Moller CH et al	10 “low-bias” RCTs, 4950 pts.	30 % higher risk of all-cause mortality after off-pump CABG compared with on-pump CABG
[28] Feng ZZ et al, Ann Thorac Surg (2009)	Meta-analysis of ten randomized trials (2,018 patients) of Off-pump surgery versus on-pump.	There was no significant difference in 1-year mortality and MACCE between the 2 procedures.
	Primary outcome was 1-year mortality and MACCE.	
[29] Tagaki H et al, J Thorac Cardiovasc Surg (2010)	Meta-analysis of RCTs regarding graft patency after off-pump versus on-pump CABG.	The results of the meta-analysis favors on-pump.
		The study concluded that off-pump surgery might increase graft occlusion by 32 %.
[30] Tagaki H et al Interact Cardiovasc Thorac Surg (2014)	A meta-analysis of RCTs for mid-term MACCE following off-pump versus on-pump coronary artery bypass grafting.	Similar mid term MACCE between the two groups.
[31] Attaran S et al, Perfusion (2014)	A meta-analysis of observational studies for Off-pump versus on-pump revascularization in females.	There was no statistical significant difference in mortality between the two groups, at 30 days.
	23313 patients (n = 9596 Off pump, 13717 On pump).	
[32] Chen YB et al, Chinese medical Journal (2012)	Forty-three randomized clinical trials were selected for meta-analysis with 8104 patients in the Off-pump group and 8724 cases in the On-pump group.	The meta-analyses suggest that Off-pump reduces the risk of postoperative AF compared with On-pump, but there is no significant difference in the incidences of stroke and MI between the two procedures.
[33] Godinho AS et al, Arquivos brasileiros de cardiologia. (2012)	Meta-analysis focused on nine randomized clinical trials, corresponding to a total of 75,086 patients.	A reduction of 18 % in the risk of cardiovascular mortality (OR: 0.82, 95%CI: 0.70 to 0.98, p = 0.03) and 27 % in the risk of stroke postoperatively (OR: 0.73, 95%CI: 0.63 to 0.85, p = 0.0001) were observed, both in favor of Off-Pump Surgery.
[34] Kuss O et al, J Thorac Cardiovasc Surg (2010)	A Meta-analysis of a total of 35 propensity score analyses was included in this study accounting for a total of 123,137 patients.	This study favors off-pump with lower mortality (odds ratio, 0.69; 95 % confidence interval, 0.60-0.75), stroke, renal failure, red blood cell transfusion (P < .0001), wound infection (P < .001), prolonged ventilation (P < .01), inotropic support (P = .02), and intraaortic balloon pump support (P = .05).
[35] Takagi H et al, Ann Thorac Surg (2010)	12 randomized trials (4,326 patients) of off-pump vs. on-pump CABG.	This study revealed a statistically significant increase in midterm all-cause mortality by a factor of 1.37 with off-pump relative to on-pump CABG (RR, 1.373; 95 % confidence interval, 1.043 to 1.808).
	This report focused on late (> or = 1 year) all-cause mortality.	
[36] Afilalo J et al, Eur Heart J (2012)	Meta analysis, Fifty-nine trials were included, encompassing 8961 patients	Similar 30-day mortality. However the incidence of post-operative stroke was reduced by 30 % on the off pump group.
[37] Sa MP et al, Rev Bras Cir Cardiovasc (2012)	Meta-analysis and meta-regression of 13,524 patients from randomized trials.	Similar 30-day mortality. However the incidence of post-operative stroke was reduced by 20.7 % on the off pump group.
	6,758 off-pump pts and 6,766 on-pump CABG pts.	
Papers with important statements, on the argument: “On versus Off Pump”		
[38] Grover F, N Engl J Med (2012)	Editorial	An interesting editorial that “reconciles” the findings of the ROOBY and CORONARY trials
[39] Puskas J et al, Innovations (2005)	ISMICS recommendations	Identifies subgroup of patients that would potentially benefit from off pump techniques.
[40] ACCF/AHA practice Guidelines, Circulation (2011)	Guidelines	The guidelines contend, both approaches are reasonable, with certain factors tilting the balance one way or the other
[41] Yadava O et al, Indian Heart J (2013)	Real world experience, with 5000 cases of off pump	Very low conversion rate and low postoperative mortality.

## Limitations

The lack of consistency of outcomes is primarily arising from the observational nature of the majority of the studies. The decision to advocate beating heart surgery has been infrequently influenced by selection bias. In some surgical series, beating-heart bypass has been preferentially adopted when good quality target vessels were present. This aspect has heavily biased results and has been heavily criticized.

Propensity matching has eliminated some of the confounding. Furthermore, some studies by using large databases were able to adjust for many important risk factors. There are 2 caveats during construction of a meta-analysis that are worth mentioned: The criticism on large meta-analysis scale reports lies on the fact that when observational studies are included then the results are skewed due to confounding factors; secondly the methodology of the meta-analysis can be appreciated in this example: if you include RCTs with no strokes you may find no significant association between off-pump and stroke rate; if you exclude RCTs with no strokes you may find off-pump to be associated with a significantly lower incidence of stroke than on-pump CABG.

It can be argued that excluding a large number of RCTs, with no reported strokes in either arm of the trial, inflates the size of the treatment effect, producing a falsely significant statistical result. Alternatively, by including zero event RCTs, the size of the treatment effect is unjustifiably diluted and this produces a falsely non-significant result. It is encouraging however that treatment effects from RCTs and propensity score analyses were very similar in a "meta-matched" population of studies; the estimated differences in "meta-odds ratios" were below an absolute value of 0.15, indicating that only a small remaining bias is present in propensity score analyses.

Finally, most trials comparing Off with On-pump surgery have excluded high-risk patients; by excluding this group from trials, the full potential of off-pump work is yet to be elucidated.

## Discussion

Extensive literature had been published on the subject of off pump coronary artery bypass surgery. With lack of robustness in the current evidence, more questions had been raised and there is currently still a controversy in its effectiveness and indication.

As a result, Off- pump surgery has fallen out of fashion in Europe and United States. Only in Japan there is consistency in off-pump practise; The Annual report of Japanese Association for Thoracic Surgery publishes high numbers of off-pump CABG performance in more than 60 % of cases, steadily over the last 10 years [38].

To summarize, we can deduce that both techniques confers similar early graft patency and in turn, similar clinical outcome up to 1 year. However, controversies still exist in terms of long-term clinical outcome and angiographic grafts patency.

The high occlusion rate of grafts in off-pump surgery must be due to high occlusion rate of saphenous veins in addition to technical difficulty. Poor graft patency and poor long-term results in off-pump surgery were related to higher venous occlusion rate because of the hypercoagulability status after surgery, in at least three reported studies [39–41].

The largest randomized control trial so far brought out more arguments than answers, by recruiting low risk patients that would be benefit by either technique. Larger meta-analyses and databases are better powered to compare outcomes and in general have shown more favourable in-hospital outcomes especially in riskier patients and equivalent long-term outcomes with off-pump compared to on-pump coronary artery bypass. Furthermore the benefits of off-pump techniques may be more apparent for patients at high risk for complications associated with the global ischemia of cardioplegic arrest and aortic manipulation.

So, who’s benefit from off-pump surgery; taken from the literature thus far, we can deduce that both techniques confers similar early graft patency and in turn, similar clinical outcome up to 1 year.

Completeness of revascularization with off pump techniques has been questioned and thus higher re-intervention rate has been reported in various studies. However one has to take into account the effect on volume-outcome relationship, which exists for morbidity and mortality after off-pump surgery with a threshold of more than 50 operations per year.

Is off-pump surgery beneficial to female gender? It may be a trend towards that but it remains to be seen.

Off pump surgery reduces the incidence of postoperative Atrial Fibrillation. This has been persistent in numerous studies being supported by robust evidence.

The current weight of evidence from systematic reviews and meta-analyses favours the position that off-pump may reduce the incidence of a stroke; Furthermore it has become apparent that non touch off-pump techniques are associated with significant reduction of the incidence of a stroke, down to less that 0.5 %.

There are numerous studies to suggest that early morbidity and mortality is reduced in high-risk patients when off-pump techniques are implemented. That may include patients with reduced EF or severe lung, renal or vascular dysfunction. Therefore, the benefits of off-pump techniques may be more apparent in patients at high risk for complications associated with the global ischemia of cardioplegic arrest and aortic manipulation.

They may be a trend towards less postoperative complications in octagenarians undergone off-pump surgery; however the up till now studies suggested that there is no survival benefit attributed to off-pump surgery.

Conversion to on-pump is associated with adverse outcome. Moreover during emergency conversion there has been observed a fivefold increased in mortality and a higher rate of postoperative complications.

The investigators from the ROOBY trial are recommending either of the two techniques based on patient factors, provided the surgeon was competent in both. For example, if a patient had renal dysfunction or a heavily calcified aorta, off pump would be preferred.

Hattler et al. reported the 1-year angiographic follow-up of the “ROOBY cohort” (AHA Congress, Orlando, 2014). The authors revealed that off-pump patients had a lower saphenous vein patency than on-pump, but a similar arterial graft patency rate, leading to less effective revascularization.

Grover [42] in an editorial tried to reconcile the difference between the ROOBY and CORONARY trial findings. The author went on to say that the CORONARY trial involved only surgeons experienced in Off-pump surgery, whereas the ROOBY trial also had trainees as operating surgeons. On a conservative note however, he cautioned that any firm conclusions would have to await long-term follow-up results.

The International Society for Minimally Invasive Cardiothoracic Surgery (ISMICS) recommendations [43] state that the use of Off-pump bypass reduces perioperative morbidity, neurocognitive dysfunction and hospital length of stay and should be considered especially in high-risk patients, for example, those with severe ascending aortic calcification, liver disease, renal insufficiency or other systemic processes that may be exacerbated by CPB, in order to reduce morbidity and mortality.

In 2011 the AHA published a balanced and sensible approach on the argument, of “On-pump versus off-pump” [44]. Based on available data, the guidelines contend, both approaches are reasonable, with certain factors tilting the balance one way or the other.

## Conclusions

In summary, although off-pump surgery, maybe produces comparable results to on pump surgery, certain groups of patients such patients with severely calcified aorta, high risk patients with severe end organ dysfunction and possibly patients undergoing hybrid revascularization procedure with PCI and Off-pump combination; and finally selected patients requiring redo surgery in an effort to potentially reduce the morbidity associated with on-pump techniques.

The “real world” experience with off-pump surgery comes from the National Heart Institute in Delhi [45] of over 5000 cases done Off-pump with an impressive conversion rate of less than 1 % and mortality of 1.6 %; this shows that off pump is an effective and safe technique, provided it is adopted and practiced properly. The best technique is that which works best for that particular patient, in the context of his clinical setting and his treating surgeon's repertoire lending credence to the belief that it is the surgeon and not the technique, which is at the heart of the problem.

As the superiority of off-pump is presently confined at high-risk patients with severe ascending aortic calcification, liver disease, and renal insufficiency, the merits of this technique is narrowed down to less than 10 % of total CABG population who really benefit from off-pump. However cardiac surgeons who are called to performed off-pump CABG surgery in less than 10 % of CABG cases (which are also technically demanding), they will be facing with a massive challenge. In order to overcome the difficulties and learning curves under stressful circumstances, daily off -pump exercise may become mandatory training so eventually the surgeon will safely perform sophisticated off pump CABG surgery such as aortic non-touch total arterial revascularization.
